# Individual and institutional capacity-building for evidence-informed health policy-making in Iran: a mix of local and global evidence

**DOI:** 10.1186/s12961-022-00816-3

**Published:** 2022-02-12

**Authors:** Leila Doshmangir, Hakimeh Mostafavi, Masoud Behzadifar, Bahareh Yazdizadeh, Haniye Sadat Sajadi, Edris Hasanpoor, Mahdi Mahdavi, Reza Majdzadeh

**Affiliations:** 1 Department of Health Policy & Management, Tabriz Health Services Management Research Center, School of Management and Medical Informatics, Tabriz University of Medical Sciences, Tabriz, Iran; 2 Social Determinants of Health Services Research Center, Tabriz University of Medical Sciences, Tabriz, Iran; 3 Health Equity Research Center, Tehran University of Medical Sciences, Tehran, Iran; 4 Social Determinants of Health Research Center, Lorestan University of Medical Sciences, Khorramabad, Iran; 5 Knowledge Utilization Research Centre, Tehran University of Medical Sciences, Tehran, Iran; 6 Knowledge Utilization Research Center, University Research & Development Center, Tehran University of Medical Sciences, Tehran, Iran; 7 Research Center for Evidence-Based Health Management, Maragheh University of Medical Sciences, Maragheh, Iran; 8 National Institute for Health Research, Tehran University of Medical Sciences, Tehran, Iran; 9 Community Based Participatory Research Center, Tehran University of Medical Sciences, Tehran, Iran

**Keywords:** Evidence, Health policy, Health system research, Policy interventions

## Abstract

**Background:**

Providing valid evidence to policy-makers is a key factor in the development of evidence-informed policy-making (EIPM). This study aims to review interventions used to promote researchers’ and knowledge-producing organizations’ knowledge and skills in the production and translation of evidence to policy-making and explore the interventions at the individual and institutional level in the Iranian health system to strengthen EIPM.

**Methods:**

The study was conducted in two main phases: a systematic review and a qualitative study. First, to conduct the systematic review, the PubMed and Scopus databases were searched. Quality appraisal was done using the Joanna Briggs Institute checklists. Second, semi-structured interviews and document review were used to collect local data. Purposive sampling was used and continued until data saturation. A qualitative content analysis approach was used for data analysis.

**Results:**

From a total of 11,514 retrieved articles, 18 papers were eligible for the analysis. Based on the global evidence, face-to-face training workshops for researchers was the most widely used intervention for strengthening researchers’ capacity regarding EIPM. Target audiences in almost all of the training programmes were researchers. Setting up joint training sessions that helped empower researchers in understanding the needs of health policy-makers had a considerable effect on strengthening EIPM. Based on the local collected evidence, the main interventions for individual and institutional capacity-building were educational and training programmes or courses related to the health system, policy-making and policy analysis, and research cycle management. To implement the individual and institutional interventions, health system planners and authorities and the community were found to have a key role as facilitating factors.

**Conclusion:**

The use of evidence-based interventions for strengthening research centres, such as training health researchers on knowledge translation and tackling institutional barriers that can prevent well-trained researchers from translating their knowledge, as well as the use of mechanisms and networks for effective interactions among policy-makers at the macro and meso (organizational) level and the research centre, will be constructive for individual and institutional capacity-building. The health system needs to strengthen its strategic capacity to facilitate an educational and training culture in order to motivate researchers in producing appropriate evidence for policy-makers.

**Supplementary Information:**

The online version contains supplementary material available at 10.1186/s12961-022-00816-3.

## Background

All governments resort to mechanisms such as public policy to solve the problems facing society, improve conditions and exploit opportunities. Various approaches have been proposed by different groups and organizations for enhancing public policy [1, 2]. One such approach that has gained momentum in the last 10 years is evidence-informed policy-making (EIPM) [3]. EIPM is an approach for policy-making that takes into account the political context, availability of resources, and people and customer experiences in order to provide the right evidence at the right time in the right language for policy-making [4, 5]. This approach enables policy-makers to make informed decisions using the best available evidence, which will ultimately improve health system performance [6].

EIPM can play a crucial role in designing, implementing and delivering better public policies and maintaining quality public services in the context of various situations.

A number of reports have provided credible information confirming that evidence produced through research can improve the health policy process by gathering valuable data regarding different phases of policy-making [7–9]. In other words, the promotion of EIPM can reduce the barriers to evidence utilization by health policy-makers [10, 11]. It has been argued that policy-makers are simply the end-users of the evidence that is produced by researchers, so it is unrealistic to expect them to incorporate and adapt all policies to local conditions [12, 13]. It is universally recognized that effective health systems that are evidence-based in their implementation are vital to improving health outcomes [14, 15].

In addition to the demand for evidence among policy-makers, the supply of appropriate evidence to policy-makers strengthens producer capacity and is a key factor in the expansion of EIPM. The increased use of evidence in policy-making means stronger capacity for evidence production on both the demand and supply side [16].

Advocates of EIPM argue that the depth and quality of knowledge used by policy-makers influence the effectiveness of policies. Necessary steps must be taken to use appropriate methods, interventions and strategies to produce the knowledge needed by policy-makers and to strengthen this policy-making approach [17]. In addition to other important factors such as the failure to correctly identify the problem or issue, and the lack of a problem-solving function that results in limited evidence-based policy-making, a lack of research-based solutions is also a critical issue. Studies and research evidence can help health policy-makers and managers evaluate current systems and design new policies and services based on their knowledge of failures and successes [18]. In addition, the use of evidence enables policy-makers to implement policies based on the best available evidence [19]. Some critics believe that there is a gap between policy-making requirements at the national level, the needs of society that must be met, and policy priorities and what motivates researchers to do research [14, 20]. On the other hand, researchers sometimes provide policy-makers with massive amounts of evidence that confounds and complicates the decision-making process.

Moreover, many countries are facing a number of barriers in terms of institutional shortcomings and gaps in knowledge and skills and capacity. In recent years, in line with the increasing global attention to the importance of evidence use in health polices, Iran’s health system has been motivated to produce interventions and initiatives towards researcher capacity-building with the aim of promoting EIPM [21].

Although in recent years various measures and interventions have been implemented in Iran's health system to strengthen and institutionalize EIPM, in reality, obtaining and using high-quality evidence is a challenging issue, and many policy decisions are still not informed by evidence [22]. Also, the use of research evidence in health policies is not systematic, comprehensive and well institutionalized [10]. In some cases, health policies are rushed and politicized without sufficient research and evidence and without scientifically getting to the root of the problem [23]. For example, based on the evidence, the development and implementation of the Health Transformation Plan, one of the largest reforms of the health system, was not based on robust evidence, and now there are many challenges in its continuity [24, 25]. In 2015, when the Sustainable Development Goals came to the fore as a political commitment for many countries, one of the goals of this very important approach was to try to eliminate viral hepatitis by 2030. However, although the neighbouring country of Afghanistan is the largest producer of narcotics, and considering the large volume of immigration into Iran and related risk of injecting drug use in young people and consequent increased incidence of hepatitis C, Iranian policy-makers still do not consider this evidence to be a major issue for hepatitis C risk in the country [26].

Each year, new issues and problems arise in the Iranian health system, with various responses from policy-makers, but many problems remain unsolved. This again reinforces the importance of EIPM and the need to institutionalize this approach in the Iranian health system [6]. In general, discussions of the practical application of evidence in health policy have been less extensive, and there has been very little discussion about Iran in the literature. Also, there is no comprehensive evidence on how to strengthen individual and institutional capacity to develop EIPM in Iran. Thus, it is necessary to strengthen mechanisms that can promote knowledge and skills among researchers and knowledge-producing organizations in establishing EIPM. In this regard, the interventions need to be focused on both the individual and institutional level. Therefore, this study aimed to review the interventions used to promote researchers’ and knowledge-producing organizations’ knowledge and skills in the production and translation of evidence to policy-making, and to explore the interventions for strengthening EIPM at the individual and institutional level in the Iranian health system.

## Methods

Review and qualitative methods were used to conduct this study, which was carried out in two phases. In the first phase, we conducted a systematic review to collect interventions on increasing individual (researchers) and institutional (knowledge-producing organizations) knowledge and skills in the production and translation of evidence to policy-making.

In the second phase, we conducted a qualitative study to explore Iranian health system experts’ viewpoints regarding necessary interventions in the Iranian health system for implementing the collected interventions to empower researchers in moving towards establishing EIPM in Iran.

### The review phase

In order to maintain the integrity of all stages of this study, the Preferred Reporting Items for Systematic Reviews and Meta-Analyses (PRISMA) guidelines were followed [27].

In response to the study question—what interventions are available to increase the knowledge and skills of researchers in producing and translating evidence needed for health policy-making—a systematic review was chosen. This type of review study helps to establish the foundations for developing evidence-informed decision-making and identifying knowledge gaps and finding topics for future research. The results of these studies have high validity due to the specific method and quality of the studies and can be beneficial for all stakeholders in the health system [28].

#### Inclusion criteria

In the context of this article, based on the research question, inclusion criteria were identified by the research team. Our inclusion criteria were as follows:

(i) Studies addressed a variety of educational programmes and interventions used to train and increase the use of EIPM in the health system.
(ii) Studies were in the English language.
(iii) Studies were published between 1992 and the end of 2019.
(iv) A variety of studies were reviewed, including systematic reviews, narrative reviews, original research, commentary, letters and editorials.
(v) Studies were published in peer-reviewed journals.

#### The literature search

Reference lists of included studies were reviewed and scanned for possible relevant studies, attempting to keep the results up to date while writing the article. Scopus and PubMed were searched. The search strategy and results for each database are listed in Additional file 1: Appendix 1 & 2. The search was carried out by two members of the research team (LD and EH). In order to increase the validity of the study, a review of articles at each stage was performed by two members of the research team (HM and EH), and the degree of agreement between them was assessed. In cases where agreement was not reached between the two researchers, a third researcher was used. In addition, during a meeting with the research team, consensus was reached on articles that were considered contradictory.

#### Data extraction

To extract the data, a researcher-designed form was used that included the most important characteristics of the papers, as follows: first author, year of publication, the purpose of the training programme, method of training, data collection, country, main findings, method for enhancing practical skills, specifications of curriculum, the content of the audience training, the credentialling organization and the curriculum evaluation.

#### Quality assessment

Due to the wide range of final studies, appropriate Joanna Briggs Institute checklists were used to evaluate the quality of the final studies. The institute has dedicated checklists for each type of study. The quality of studies was classified into three categories: good, moderate and poor.

### The qualitative phase

The COREQ (Consolidated Criteria for Reporting Qualitative Research) checklist was followed for the qualitative reporting phase. In this qualitative study, data were collected through interview and documentary review. Semi-structured interviews were used to collect the opinions and experiences of key health experts regarding capacity-building among researchers and knowledge-producing organizations in the production and translation of evidence required for policy-makers and providing appropriate empowerment programmes in Iran.

#### Study participants and data collection

Participants including researchers, health policy-makers, health planners and faculty members were selected from various institutions through purposive sampling. Documents were also selected from various relevant organizations including health research centres and institutes, faculties and health broker organizations. Purposive sampling was conducted using the maximum diversity method to select research participants. Sampling was continued to reach saturation. Inclusion criteria for participant selection included individuals with experience in EIPM such as publishing papers, reports or projects. Interviews were conducted in the participants’ office or the place that they preferred. Before starting the interview, informed consent forms were completed by the participants. We assured participants that they could exit the study at any time. Interviews were conducted based on an interview guide which we developed according to the study objectives covering two types of information including demographic questions and the main questions of the study. To finalize the interview guide, three preliminary interviews were conducted. The interview guide is provided in Additional file 2: Appendix 2. Four interviews were conducted by sending an email to the participants and asking them to write their answers on the paper or record their answers to the questions.

#### Data analysis

Each interview was transcribed verbatim. To improve the rigour of the study, various activities were carried out. To increase credibility, dependability, conformability and triangulation, we used several resources for data collection, including conducting interviews with various participants and conducting document reviews. After interviews, transcribed files were sent to participants for confirmation or to make any changes they wanted. To increase transferability, purposive sampling was used. Data were analysed according to a five-stage thematic framework approach, including familiarization, identifying a thematic framework, indexing, charting, mapping and interpretation.

## Results

The results of our study are provided in two main parts: the review phase results and the qualitative phase results.

### The review phase

**Fig. 1:**
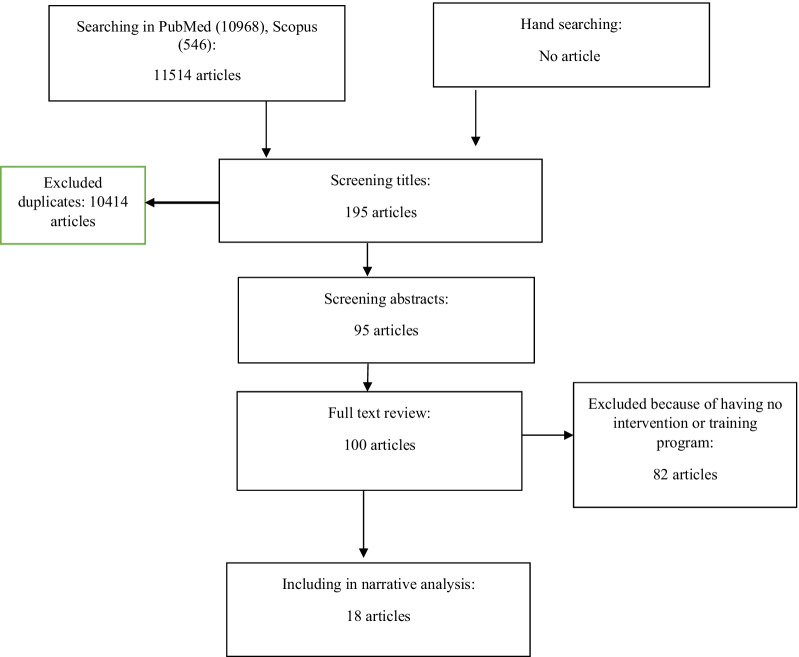
Selection of studies for review analysis

Our search yielded 11,514 articles, 10,414 of which remained after deleting the duplicates. Following the secondary screening and examination of the articles’ full texts, 18 articles remained. The PRISMA flowchart of the study’s articles is illustrated in Fig. 1.

The characteristics of the included studies are presented in Tables 1 and 2. Most of the studies were from Nigeria, among which six were by one author and his colleagues. Other studies were from Canada (three studies), Australia (two studies), Mexico, England, Fiji, Hungary, Belgium, South Africa and the European Union. Interventions were performed over periods ranging from 1 day to 3 years. In the study by Uneke et al. in 2012, a 1-day workshop was used for training [29], while in the study by Dreisinger et al. in 2008, online training was conducted from 2001 to 2004 [30]. The studies reviewed were published in different journals. The largest number were in *Health Research Policy and Systems*, with three studies published. The other journals included the *Pan American Journal of Public Health*, *International Journal of Technology Assessment in Health Care*, *Healthcare Management Forum*, *BMC Public Health*, *Evidence & Policy*, *Energy Strategy Reviews*, *Public Health*, *Global Public Health*, *Journal of Health Services Research & Policy*, and *Health Policy*.

**Table 1:** Characteristics of included studies

Authors (year)	Objectives	Country	Study design	Participants (*n*)	Data collection and analysis	Contexts	Methods of intervention	Curriculum specifications	Training programme content	Funding organization	Outcomes	Evaluation results	Quality
Uneke et al. (2015) [31]	To increase the interaction among researchers, policy-makers and stakeholders in decision-making	Nigeria	Evaluation of the implementation of a series of interventions by an implementation research framework	Ten policy-makers and researchers from health ministry and university	Interviews Giorgi’s phenomenological approach	Ministry of Health	Twice-weekly training sessions at Ebonyi State University, Nigeria, and Ministry of Health for 1-day policy briefing, policy workshop, policy dialogue between policy-makers and researchers	Policy-makers were involved in research and educational activities, such as working on the research ethics committee. Researchers also played an advisory role in the policy-making processes of the Ministry of Health	Policy-makers participated in university research activities; at the end of the 6-month period they were asked to write a brief policy on malaria based on what they learned	WHO and International Development Research Centre	Improvement in knowledge of evidence-to-policy, mutual mistrust between policy-makers and researchers, and the awareness of importance of the health policy advisory committee (HPAC)	Evaluation results showed a noteworthy improvement in knowledge of evidence-to-policy link among the HPAC members, the elimination of mutual mistrust between policy-makers and researchers, and an increase in the awareness of importance of HPAC	Good
Ju et al. (2014) [32]	To evaluate the new health technologies and selection of necessary technologies	Australia	Mix of qualitative and quantitative methods	Members of the Health Policy Advisory Committee for Health Technologies Evaluation	Standardized memoranda of understanding Cost minimization analysis	Queensland Department of Health	Newsletters, presentations, posters, flyers, health technology assessment website	Teaching tools to evaluate health technologies and the need to purchase and distribute them nationally	Skills to evaluate health technologies; a qualified secretary with interdisciplinary knowledge was trained by committee members to carefully select the technologies needed in the health sector (health technology assessment programme)	Queensland Department of Health	Organizational and economic feasibility of adopting the technology, its effectiveness, implementation of QPACT (Queensland Policy and Advisory Committee for new Technology) recommendations	Evaluation of the training provided to the committee showed that of the 34 technologies requested, only 17 were deemed necessary, 7 were rejected, and the rest required further consideration. It confirmed the effectiveness of the training provided to committee members	Good
Khan et al. (2014) [33]	To facilitate the collaboration between the Ontario Drug Policy Research Network and its policy-maker centre	Canada	Rapid pharmaco-epidemiological research	Policy-makers at the Ontario Ministry of Health and researchers	Healthcare services data for the entire population of Ontario Rapid analyses	Ontario Ministry of Health and Long-Term Care (MOHLTC) Drug Innovation Fund and the Institute for Clinical Evaluative Sciences (now ICES)	Regular meetings, theoretical topics, case study of “hypertension drug therapy”	Conducting the monthly meetings and rewriting research questions, analysing and collecting relevant data, effectively disseminate research results	Teaching how to rewrite research questions, analyse and collect relevant data, effectively inform research results	Ontario MOHLTC Drug Innovation Fund and ICES, a nonprofit research institute sponsored by the Ontario MOHLTC	Partnerships between researchers and policy-makers	Collaborative research processes can produce mutually beneficial partnerships between researchers and policy-makers. Effective integration of research into policy-making will lead to informed decisions that profoundly affect the well-being of society at large	Good
Langlois et al. (2016) [34]	To increase policy-makers' participation in real-world decision-making and policy-making and to encourage interaction between researchers and policy-makers	Mexico, Nicaragua, South Africa and Cameroon	A mixed-methods realist evaluation design	Healthcare professionals, health system stakeholders, researchers (*n* = 221)	In-depth interviews and a focus group discussion, thematic content analysis, descriptive	Maternal health programme management teams and implementers in selected regions of Mexico and Nicaragua	Workshops, theoretical education, case study of maternal health programme	Programme implemented in three Mexican provinces and three Nicaraguan departments May 2013–March 2015, Policy Building Demand for evidence in Decision making through Interaction and Enhancing Skills (BUDDIES)	Teaching the importance of strengthening the interaction between researchers and health personnel at national and provincial levels and promoting policy-makers' participation in research	Implementation research platform, Alliance for Health Policy and Systems Research, Norwegian Government Agency for Development Cooperation, Swedish International Development Cooperation Agency, United Kingdom Department for International Development	Implementation research, quality of maternal healthcare programmes, health programme personnel, capacities to identify and use evidence, medication adherence for chronic diseases, policy-makers engaged in “buddying” process, recognition of the value of research, and greater demand for policy-relevant knowledge	To evaluate the second programme, national documents were analysed, technical reports were analysed, and message exchange was conducted between researchers and policy-makers, and in-depth interviews were conducted with policy-makers and educators and researchers	Good
Lavis et al. (2012) [35]	To help policy-makers in formulating appropriate policy briefs for health challenges	Burkina Faso, Cameroon, Mozambique, Zambia, Central Africa, Ethiopia	Qualitative	Policy-makers and researchers from six African countries	NR	Local, district, national levels	Conducting workshops to train how to formulate a policy brief	The workshop gave tips on how to include services, financing the malaria programme, how to provide and distribute medication to patients, and how to diagnose the disease in the malaria policy brief	The workshop gave tips on how to fund a malaria programme, how to provide and distribute medication to patients, and how to diagnose the disease in the malaria policy brief	Canada Research Chair in Knowledge Transfer and Exchange	Supporting evidence-informed health systems, feedback on the approach, understanding how to match any given knowledge translation platform’s infrastructure, activities and outputs to particular contexts to achieve the greatest outcomes	It is supposed that policy-makers use the policy briefs as key data in their policy dialogues	Good
Lefebre (2010) [36]	To integrate the evidence used in senior- and mid-level management	Canada	A case study approach using mixed methods	Mid-level and senior managers of a home healthcare organization	Dobbins framework, the Canadian Health Services Research Foundation (CHSRF) self-assessment tool, and a management interview tool	A home healthcare organization (Saint Elizabeth Health Care)	Discussion sessions, workshops	Workshops and training sessions over an 18-month period by experts and professors in the field of knowledge transfer and uptake	Identifying a number of key organizational strategies, discussing them, identifying barriers between senior and mid-level managers, communicating with other organizations to empower managers	EXTRA fellowship programme	Changes in managers' behaviour and use of scientific evidence in decisions	One year after the implementation of the programme, its effectiveness was assessed. Evaluation results indicated changes in managers’ behaviour and use of scientific evidence in their decisions	Good
Mavoa et al. (2012) [37]	To develop and evaluate a knowledge-mediated approach to formulating obesity reduction policies in Fiji	Fiji	A multistage method—qualitative (This is a study protocol)	Key people from healthcare organizations, agriculture and educational organizations	Interviews, structured questions, themes and constant comparative analysis	Public health and community-based obesity prevention	Conducting workshops to teach evidence-based policy-making	A set of workshops would be conducted for each of the organizations	These workshops would cover policy and policy cycles, the policy environment, the definition of evidence, the sources of evidence and knowledge brokering	Fiji National University, Fiji Ministry of Health and Deakin University	The effectiveness of a knowledge-brokering approach	NR	Moderate
Makkar et al. (2016) [38]	To measure the impact of interventions on the rate of web using among policy-makers	Australia	Time series study	Employees (*n* = 392) of various state and federal agencies (*n* = 5)	Email and website Time series analysis	Australian policy-making organizations (*n* = 97)	Web CIPHER tool	The five institutions with the most applicants were selected. The intervention began in November 2014 and continued through February 2015. Searches for each institution were monitored for 1 month. During the first 2 weeks of the month, educational material was posted to the institution’s website	During the first 2 weeks of each month, educational materials were posted on the corporate website. Then, the time series method was used to measure the amount of employees' use of submissions and the impact of training content on performance and decisions. The autoregressive integrated moving average (ARIMA) model was used to illustrate the change in the use of educational materials	Australian National Health and Medical Research Council Centre of Research Excellence	Number of articles and blogs on usage by all members, Web CIPHER tool usage across all member organizations, tailored articles on usage, tailored blogs on usage, usage by the target agencies, tailored articles on targeted agencies’ usage, blogs on targeted agencies’ usage	The ARIMA model was used to illustrate the change in the use of educational materials. The results showed that studying the articles on the web was increased by the staff	Good
Ouimet et al. (2015) [39]	To evaluate the impact of an evidence-based policy-making course on students’ knowledge	Canada	A controlled before-and-after design	Master of science students in political science Control (*n* = 13) Treatment (*n* = 13)	Questionnaire Multivariate regression analysis	Université Laval’s Master’s Programme in Public Affairs, Université Laval’s Master’s Programme in Political Science	Theoretical training, training group and control group were selected among students. One group was trained in evidence-based policy-making and the other group was trained in research methodology	Before taking the course, both groups underwent tests. Then both groups participated in a mandatory 45-hour training course. Courses were conducted in the winter term	Intervention group was educated about different types of research methods, systematic review and critical evaluation. In the control group, a systematic review of the research methodology was taught. At the end, the test was taken in both groups	Political Science Department of Laval University	Basic knowledge for literature search, awareness of the existence of risk of bias in scientific studies, basic knowledge of key research designs, other basic methodological knowledge	Comparison of pre- and post- test results showed that the knowledge level of the intervention group increased	Good
Uneke et al. (2017) [40]	To improve the knowledge and capacity of policy-makers and research team of maternal and child health programme using knowledge translation programmes and Equitable Impact Sensitive Tool (EQUIST)	Nigeria	A modified before-and-after intervention study	Members of International Development Research Centre/West African Health Organization supported implementation research team of Edo state; policy-makers from Edo State Ministry of Health and related agencies; staff from local government area health departments, staff of the Primary Health Care Development Agency (PHCDA), and representatives of the civil society organizations/nongovernmental organizations (CSOs/NGOs) as well as media representatives	Questionnaire Analysis: using the methods developed at McMaster University Canada by Johnson and Lavis	Edo State Ministry of Health and related agencies, local government area health departments, PHCDA, and representatives of the CSOs/NGOs	Workshops by policy-makers and research team of the maternal and child health programme (*n* = 45)	A 3-day workshop on knowledge translation and EQUIST was conducted. At the beginning and end of the course, a test was conducted	Knowledge translation templates, tools and indicators, policy review, policy formulation, position review, scenario analysis	International Development Research Centre of Canada	Knowledge translation tools, intersectoral collaboration in policy-making and implementation, managing political interference in policy-making and implementation, policy review, analysis and contextualization, policy formulation and legislation process, introduction to MBB (marginal budgeting for bottlenecks tool): its weakness and replacement by EQUIST, EQUIST overview and theory of change	Test results at the end of the workshop showed that the level of knowledge of policy-makers had increased	Good
White et al. (2018) [41]	To advise the South African national tuberculosis (TB) programme managers on evidence-based policy-making 2014–2016	South Africa	NR	National TB programme financing providers and other key stakeholders	Documentary evidence, interviews and direct observation	The South African government health and finance departments	Meetings, modelling and telephone calls	In addition to answering audience questions, training was provided on information and communication technologies (ICTs), budget forecasting, monitoring of new policy implementation, new human resources (HR) programmes	In addition to answering questions, training was provided on ICTs, budget forecasting, monitoring of new policy implementation, new HR programmes and nurturing	United Kingdom Medical Research Council, United Kingdom Department for International Development, the Bill and Melinda Gates Foundation, Unitaid	An “institution”, a “policy dialogue forum” and an “interface”	Analysis of evidence documents, interview and direct observation were used for evaluation. The results showed the positive effect of training on the use of evidence in decision-making	Good
Dreisinger et al. (2008) [30]	To determine the impact the course had on the implementation of evidence-based public health (EBPH) within their respective public health agencies	USA	An online evaluation of the course	Programme managers, assistant directors, programme coordinators, programme directors and programme chiefs (*n* = 107)	A 15-question web-based survey (email), descriptive statistics (mean, median, standard deviation)	Respective public health agencies (state health departments)	A training course to enhance evidence-based decision-making	Ability to utilize the course content. This included factors external to the course content (e.g. time constraints at work) and factors more directly related to the course content (e.g. time constraints during the course), lack of relevance, complex information, and cumbersome binder	Questions were framed to determine the perceived helpfulness of the course content (i.e. whether it helped the respondent improve specific competencies or skills)	National Association of Chronic Disease Directors, the Directors of Health Promotion and Education, and the Missouri Department of Health and Senior Services	Referred to the EBPH texts, used the EBPH materials in planning a new programme, in modifying an existing programme, for grant applications, in searching the scientific literature for information on programmes, in evaluating a programme, to develop a rationale for a policy change, to convey the economic impact of a programme or policy, to prepare a policy briefing for administrators or state or local legislative officials, taught others how to use/apply the information in the EBPH course and to design and deliver an EBPH course of their own	Results from the evaluation revealed that 90% of participants indicated that the course helped them make more informed decisions in the workplace. Respondents identified improvement in their ability to communicate with their coworkers and read reports	Moderate
Waqa et al. (2013) [42]	To empower policy-makers for formulating evidence-based policies to improve nutrition and physical activity	Fiji	Mix of qualitative and quantitative methods	High-level officers (*n* = 49)	Semi-structured interviews, questionnaire	Government agencies and NGOs	Four to six independent workshops for six projects; each workshop lasted 2–3 hours	Some of the components of the policy, the policy cycle, how to access the evidence and scientific and research information, how to use evidence in policy-making were taught	Governmental agencies and NGOs were invited to participate in the project, then one participant was selected from each organization. Finally, workshops were conducted	Australian Agency for International Development (AusAID) on an Australian Development Research Awards grant	Active participation of knowledge brokers, skills in EIPM and policy development, EIPM capacity	Active participation of the members in the workshops, formulation of policy briefs on the basis of the lessons learned showed that the workshops were effective	Good
Waters et al. (2011) [43]	To identify feasible, acceptable and ideally, effective knowledge translation strategies to increase evidence-informed decision-making in local governments	Australia and New Zealand	A cluster-randomized controlled trial	The decision-makers	Telephone calls, analytical statistics	Victorian local governments (*n* = 45)	Workshop	Access to research evidence, critical evaluation tools	The programme would run for 2 years. Weekly training sessions were conducted for participants	National Health and Medical Research Council of Australia	Access to research evidence, confidence using research evidence, organizational culture for evidence-informed decision-making, influence of research evidence on public health decisions, usefulness of research evidence in decisions	NR	Moderate
Uneke et al. (2017) [44]	To reduce the gap between researchers and policy-makers in order to facilitate evidence-based decision-making and policy-making according to national health priorities	Nigeria	An exploratory investigation with a quantitative cross-sectional survey technique	National and local health policy-makers and decision-makers	Questionnaire, descriptive statistics	Ebonyi State Ministry of Health (ESMoH)	Workshops	Evaluation of the six policy briefs about evidence and information, financing, service delivery, medical technologies and products, health workforce, governance and leadership	Understand policy-makers’ evidence need, play expert advisory role and provide scientific evidence to guide policy issues, provide capacity enhancement for policy-makers	United Nations Children's Fund (UNCICEF)/United Nations Development Programme (UNDP)/World Bank/WHO Special Programme for Research and Training in Tropical Diseases	Researcher and policy-maker collaboration in EIPM	The outcome of this study clearly suggests that secondment has great potential in promoting EIPM and merits further consideration	Good
Uneke et al. (2012) [29]	To increase the capacity of the health policy advisory committee and empower the committee members with the necessary skills for evidence-based decision-making and knowledge translation	Conducting the implementation of the health policy advisory committee	Mix of qualitative and quantitative methods	Directors from the health ministry (*n* = 9), senior researchers (*n* = 5), a director in the local government service commission, and two executive directors of health-based NGOs (*n* = 2)	Six workshops and face-to-face discussions policy dialogues	Ebonyi State Health Policy Advisory Committee (ESHPAC)	Face-to-face training	Advisory committee members, national and local health policy-makers and stakeholders, decision-makers, researchers	Using the support tool and applying the evidence-based networking, empowerment of assistant professors in a 3-month programme by the state university, developing a policy brief to improve government performance on maternal and child care programmes, implementing the policy dialogue between policy-makers and other stakeholders related to maternal and child care programmes	WHO Alliance for Health Policy and Systems Research		The results of the training evaluations showed that decision-makers had increased awareness of the importance of using evidence to promote the use of research evidence at the Ministry of Health. The gap between researchers and policy-makers was also narrowed	Moderate

*NR* not reported

**Table 2:** Characteristics of included systematic reviews

Authors (year)	Objectives	Country	Study design	Included papers (*n*)	Analysis	Databases searched	Population	Interventions/phenomena of interest	Comparison/Context	Outcomes	Funding organization	Main results
Innvaer et al. (2002) [45]	To summarize the evidence from interview studies regarding facilitators of and barriers to the use of research evidence by health policy-makers	Norway	A systematic review	24	Descriptive and qualitative	MEDLINE, Embase, SocioŽ le, PsychLit, PAIS, IBSS, IPSA and HealthStar	Health policy-makers	Health policy-makers’ perceptions of their use of evidence	International	Use of research evidence	NR	Interview studies with health policy-makers provide only limited support for commonly held beliefs about facilitators of and barriers to their use of evidence, and raise questions about common-sense proposals for improving the use of research for policy decisions. Two-way personal communication, the most common suggestion, may improve the appropriate use of research evidence, but it might also promote selective (inappropriate) use of research evidence
Uneke et al. (2017) [14]	To assess the efforts and various initiatives that have been undertaken to deliberately engage policy-makers and other stakeholders in the health sector in Nigeria for the promotion of EIPM	Nigeria	A systematic review	11	Descriptive	MEDLINE (PubMed)	Researchers and policy-makers	Workshops	Health sector in Nigeria	An assessment of policy-makers’ engagement in initiatives to promote EIPM	International Development Research Centre Canada	All the studies indicated positive outcomes and impacts in relation to quantifiable improvement in policy-makers' knowledge and competence in evidence-to-policy process

*NR* not reported

Most studies used a mix of quantitative and qualitative methods [31, 34, 46] for data collection and analysis. Studies showed that a series of interventions including workshops [14, 47], online training [30, 48] discussion sessions, email training [43, 49] and websites were used to train EIPM. In most cases, questionnaires were used as pre- and post-tests to compare the effectiveness of educational interventions.

In some studies, to assess the impact of the intervention on researchers’ and policy-makers’ knowledge, their performance was reviewed at the end of the course to quantify their use of scientific evidence in their day-to-day practical decisions as well as their ability to formulate policy briefs. Evaluation of the results of the interventions in all studies showed a positive effect of the interventions on increasing the knowledge of the participants in the educational courses.

### The qualitative phase

In total, we conducted 18 interviews. Table 3 shows the positions and characteristics of the participants. The interventions for capacity-building to strengthening EIPM were provided at the individual and institutional levels (Table 4).

**Table 3:** Characteristics of interviewees

Organization	Number of participants	Sex		Academic discipline	
		Male	Female	Health policy	Other disciplines
Health research centres	2	0	2	1	1
Health research institutes	2	1	1	0	2
Research units of the Ministry of Health and Medical Education	4	1	3	2	2
University of medical sciences	13	3	7	5	5
Total	18	5	13	9	9

**Table 4:** Themes and subthemes of interventions for individual and institutional capacity-building to strengthen EIPM

Theme	Subtheme
Institutional interventions	Training
	Knowledge management and organizational communication management
	Research cycle management
	Assessment and evaluation
	Culture change
	Knowledge of policy-making and policy analysis
Individual interventions	Behavioural-motivational interventions
	Knowledge of policy evidence production and translation
	Knowledge of scientific communication
	Knowledge of the health system

#### Institutional interventions

Institutional interventions are categorized into: training programmes, knowledge management and institutional communication management, and research cycle management.

#### Training/courses for researchers

Review of the documents and analysis of interviews indicated that one of the most important measures for capacity-building among researchers and knowledge-producing organizations is holding relevant specialized training programmes or courses.

According to some of the participants, the use of short-term specialized training programmes which can be defined at the institutional level is a highly effective educational method. The nature of these programmes and the course subjects and content will help researchers and others involved in knowledge production to develop the required skills allowing them to meet the needs of policy-makers. In most cases, short-term training programmes are designed and implemented for two main reasons: to teach scientific and technical skills and to change organizational behaviour.

It's necessary to assess the needs of the consumers of knowledge products and develop training programmes accordingly. (interviewee 9 [I-9])

The use of modern teaching techniques such as distance education and designing and planning the training programmes based on policy-makers' expectations were other issues raised by the study participants.

#### Knowledge management and organizational communication management

According to the interviewees, in the context of EIPM, knowledge management is a systematic approach to understanding and using knowledge or information and making it available to policy-makers at the right time, thus allowing them to make informed decisions. The findings of the present research indicate that achieving EIPM requires knowledge management to be focused on projects and knowledge development, establishing the relevant knowledge base, the exchange and sharing of knowledge among the employees of the organization, and training.

There should be a system in place for registering policy researchers and monitoring their scientific and professional activities. (I-1)

#### Research cycle management

Effective research cycle management is an important consideration in interventions for empowering researchers to strengthen EIPM. The research cycle begins with the proposal of a research idea and design, and continues until evidence production and application. By focusing on the production of credible evidence, effective research cycle management leads to the identification of more influential and higher-quality research and lays the groundwork for better use of evidence by users such as policy-makers.

It’s especially important to pay attention to the quality of data produced in organizations. (I-12)

The findings indicated that research cycle management emphasizes the adoption of strategies for directing knowledge creation towards more influential and higher-quality research and preventing the unhindered publication of “pseudo-research”.

Evidence production networks in the country are faced with poor management. For example, research priorities in the networks of the Ministry of Health and the way these priorities are established are unclear. (I-14)

#### Culture change

The interviewees believed that evidence-based health interventions and policies must be promoted and accepted by all stakeholders. If this becomes the prevailing practice, it will lessen the impact of conflicts of interest and political inclinations on decision-making.

We all know the significant impact and role of pressure groups in the world of policies. We must force policy-making toward demanding appropriate evidence from researchers and research centres. Once we build this culture, researchers and knowledge producers will also have to improve to keep pace with policy-makers' demands. (I-7)

#### Evaluation and assessment

According to many of the interviewees, changing the evaluation and assessment system, defining motivators, changing the reward system, especially for faculty members and researchers with respect to policy-focused knowledge translation, and changing researchers ranking method in the Research Department of the Ministry of Health play a significant role in the promotion of this policy approach.

An issue that must be addressed is the evaluation system. Faculty members and students are only concerned with producing articles, not being responsive to the needs of different groups and the society as a whole. (I-10)

#### Individual interventions

##### Behavioural-motivational interventions

Motivational interventions play a key role in the performance of researchers and knowledge-producing organizations and are critical to strengthening EIPM. Knowledge about the personal characteristics of individuals in an organization and the use of various motivators will enable them to communicate more effectively with their audience and strengthen this policy approach. Designing the educational courses based on researchers’ needs, asking them to present their personal interests in research fields, and asking them to assume a main role in the training courses can motive them to act beyond what is expected.

As a researcher, I would like to be given the opportunity to study in other countries, because it will increase my knowledge, and as a result I will be able to do better researches on issues related to the health system. (I-7)

Moreover, demonstrating the key role of researchers in developing the organization’s plans will motivate them in future activities.

We as researchers expect to be educated according [to] our needs, we want to know about the complicated entity of the health systems, and we also like to know what exactly health policy-makers expect of us. We want to have a common language to have a better [understanding] of community’s needs. (I-10)

According to many of the participants, multidimensional behavioural-motivational interventions have a significant effect on individual performance.

It may seem strange to you, but sometimes giving a small gift or even a credit card increases our motivation to participate in training courses. (I-13)

##### Knowledge of policy-making and policy analysis

According to the interviewees, one of the issues that is often ignored with regard to strengthening EIPM is the considerable gap between the world of research and the world of policy-making, which acts as a major barrier to expanding this policy approach. To reduce this gap, researchers and faculty members need to become familiar with the basic concepts and principles of policy-making and policy analysis and apply them in their research endeavours.

Individuals and organizations that seek evidence-informed policy-making must possess both policy-making and evidence-related knowledge. (I-3)

The participants believed that problem for science and evidence-based policy comes when politicians and other political actors decide to discredit the science on which a conclusion is based or bend the science to support their policy position. This is called policy-based evidence as opposed to evidence-based policy. Researchers who possess the knowledge of policy-making can prevent this and use policy-makers’ language to promote EIPM.

##### Knowledge of policy evidence production and translation

The interviewees were of the opinion that the gap between the knowledge produced by researchers and organizations and the decisions and policies made by senior officials is a key challenge in the implementation of EIPM. Interventions are needed to bridge this gap, including the provision of skills and knowledge of policy-related evidence production and translation. An understanding of knowledge translation was highlighted by the participants as a key factor in strengthening the adoption and application of research results by policy-makers, which will enable them to make informed, knowledge-based decisions and policies.

Instruments that can contribute to informed policy-making and decision-making and translating evidence into the language of policy-makers aren’t used extensively. Policy-makers don't receive the necessary skills. Employees almost never reach the level of knowledge translation in evidence-informed policy-making and remain at the level of producing articles, mostly at the national level. (I-5)

##### Knowledge of science communication

Communicating science to policy-makers is one of the skills that was underscored by the interviewees. They argued that science communication must be in both oral and written format. The use of instruments in the language of policy-makers can attract their attention to a specific problem and prompt them to implement appropriate interventions.

In our country, there is a weak link between knowledge producers and policy-makers. Each policy-maker must be guided by a number of researchers. We can argue that these two groups are still in dire need of better communication. (I-14)

According to the participants, familiarity with the characteristics of research data users (i.e. policy-makers and decision-makers) and the nature of their work allows for establishing more effective science communication, conducting research with the participation of decision-makers, and identifying the social barriers to changing evidence users’ behaviour.

##### Knowledge of the health system

To institutionalize and strengthen EIPM, researchers and knowledge-producing organizations need to understand and be aware of the functions and goals of the health system, its different sections and components, how it affects and is affected by other sectors, and the history and outcomes of important actions and policies implemented within the health system. Some interviewees believed that issues such as systems thinking and systems dynamics can also be influential in a better understanding of the outcomes and effects observed within the health system and using this knowledge to inform policies.

## Discussion

The institutionalization of EIPM in any country is a complex and complicated process and requires various initiatives both among policy-makers (pull side) and researchers (push side) and in the health system and the health system policies and plans [6, 22]. This study focused on developing evidence-based and context-aware policy interventions for increasing the capacity of individual and institutional capacity-building for EIPM in Iran.

Developed countries have a long history of policy-making based on evidence, but a review of the studies showed that even countries such as Canada, England and Belgium have continuing programmes for training and updating health policy-makers in order to apply evidence in decision-making. Studies showed that one of the main dreams of researchers is to increase the application of evidence in decisions and policies. To make this dream come true, they have been conducting various interventions at the individual and institutional level.

One of the best means for capacity-building to strengthen EIPM is to conduct training courses using various methods. Training is a key step in developing a skilled workforce and a useful tool for improving the quality of policy-related research. Formal education in universities (e.g. through specifically designed courses and/or by including relevant discussions in other courses), shorter training programmes, workshops, and on-the-job training are some of the suggestions made by the participants.

According to the findings of our review, face-to-face workshops and policy briefing were the most commonly used interventional method. A possible explanation for this might be that the interactional nature of workshops makes them a popular method for training, especially in sessions with different groups of audiences. Also, this result may reflect the fact that the possibility for direct discussion and participation in topics accelerates learning by the audience.

Online training through a website or sending the educational material by email was the second most effective method. In this way, participants who could not participate in sessions at a specific time and place could benefit from online training. Moreover, the low costs and ease of online training could be a reason for the popularity of virtual training [38]. However, this method is limited for organizations with poor internet access, so high-connectivity internet must be available for all participants. The university course over several months that was held as an academic course was the third most common method. These courses are useful, but it seems that developing a suitable long-term programme, adequate budget and interested audiences are essential for successful conduction of the training [50]. Of course, online implementation of these courses can allow researchers and policy-makers in low- and middle-income countries (LMICs) to spend less time and money while obtaining useful knowledge about research and policy issues. However, one of the best ways for increasing audience empowerment and knowledge is to conduct training courses through diverse methods [51]. It seems that applying several methods increases the influence of training [52].

The results of the interviews showed that the combination of knowledge management and organizational communication management is a good option for presenting and disseminating the best and most appropriate knowledge within the organization. Training is an essential element and an indispensable part of the human development activity in this regard. Our findings indicated that providing researchers and knowledge-producing organizations with relevant training will help improve their attitudes and enhance their knowledge and skills, thus playing a key role in strengthening EIPM. Uneke et al. believe that holding training courses is a major step towards bridging the gap between research and practice and strengthening EIPM [47, 53]. Research management in the field of medical science requires managing and coordinating research activities, determining research priorities, formulating strategies and policies, and managing information [54].

Knowledge management and organizational communication management optimize the relationship between an organization or firm and its customers, partners and suppliers to maximize opportunities [48, 55]. Production of knowledge and science is the main mission of universities and research centres, and this can only be achieved through research. However, it must be noted that being evidence-based is an essential characteristic of modern science. Research in the area of health policy-making is a systematic process for knowledge production to enhance the functions of the health system, including service provision, resource generation, financing and stewardship, and contributes to attaining its goals and ultimate objective, which is to enable all people to achieve their fullest health potential [15, 56].

Today, in order to ensure more successful implementation of EIPM, knowledge-producing organizations must pay special attention to knowledge management and organizational communication management, and must increase their communication with their audience, including decision-makers and policy-makers. Our findings highlight the importance of providing the right knowledge at the right time, that is, at the point of decision-making, by implementing knowledge management in healthcare organizations. Shahmoradi et al. [57] argue that it is very important to use appropriate tools and user-friendly systems for knowledge management, as this can significantly improve the quality of decisions made in various organizations [58, 59].

Issues related to research cycle management include developing strategies aimed at continuous and constructive cooperation for strengthening EIPM among knowledge producers (including researchers and knowledge-producing organizations) and knowledge users, providing support for policy-related data collection, archival, maintenance and distribution, and facilitating access to relevant data by users such researchers.

Researchers play a key role in exploitation of knowledge. Therefore, empowering them in knowledge exploitation activities is imperative [60]. A key requirement in these initiatives is taking measures for disseminating the knowledge obtained from research [61]. In this regard, one of the necessary interventions is to enhance the understanding of knowledge translation in this group. Knowledge translation includes a wide range of activities that can be used to effectively convey the message of the research to the target group (in this case, the policy-makers) [62, 63]. Researchers sometimes tend to overestimate their knowledge translation activities, so it is necessary to train and familiarize them with all the aspects of knowledge transfer and translation as well as the methods for evaluating these activities, which can be highly effective in operationalization and use of research results [64]. Some studies showed that many researchers only seek to enhance the level of awareness among research audiences and stakeholders by relying on passive dissemination of knowledge, while paying little attention to changing users' performance and behaviour as the main goal of knowledge translation [19]. The findings indicate that strengthening EIPM requires researchers to continuously provide policy-makers with appropriate evidence-based insights and scientific advice to help them in their decision-making. However, a serious gap still exists in Iran, where researchers’ recommendations do not produce desirable results, resulting in the adoption of inefficient and ineffective policies that fail to solve the general problems faced by the society and the health system.

Suggestions for the successful communication and operationalization of policy recommendations include the acquisition of knowledge related to policy-making and the translation of knowledge, including how to formulate and publish policy briefs or hold policy discussions, and how to identify and clarify policy problems [65–68]. Science communication is an important intervention for strengthening EIPM and was highlighted by many of the interviewees. It must be noted that science communication is not limited to empirical science, but also includes nonempirical reasoning (e.g. philosophy, techniques, and skills) [69]. The former can be acquired and is transferrable, but the latter is a larger part of knowledge that is intuitive and cannot be acquired. This form of knowledge requires special individual abilities and cannot be transferred to others [70, 71].

It appears that due to the absence of information on the topic of EIPM-related knowledge production and translation, there is little evidence on transferring tacit knowledge and converting it to explicit knowledge. Therefore, interventions for increasing the knowledge and skills of researchers and allowing them to strengthen EIPM have received ever-increasing attention in recent years.

## Study strengths and limitations

The use of the two methods and collecting local and global evidence provided an in-depth view of the interventions for EIPM capacity-building. In addition, the level of detail that we found in qualitative research about interventions at the individual, institutional and health system levels is valuable. Some limitations need to be considered when interpreting the findings. Although the main focus of the review results is on the training and educational interventions at the individual and institutional levels, in the qualitative part of the study we also explored interventions at the community and the health system level. Due to a lack of sufficient evidence on the priorities of the proposed and implemented interventions for individual and institutional capacity-building, we tried to include all interventions that were effective.

## Conclusion

Incompatibility of health policy decisions with scientific evidence highlights the importance of creating a common language between policy-makers and researchers. It is necessary to set up different types of training sessions that empower the researchers for understanding the needs of health policy-makers to appropriate evidence. International organizations’ support could facilitate conducting useful training programmes. Developing evidence based and practical interventions to improve individual and institutional capacity for EIPM such as conducting training programmes or courses for researchers, using mechanisms and networks for effective interaction and cooperation among knowledge producers and knowledge users or relying on incentives that encourage individuals and organizations to be more involved in conducting evidence based research and translating research knowledge into policy can be constructive in this regard.

Generally, to facilitate better use of evidence by policy-makers, EIPM needs be strengthened in a systematic way through initiatives aimed at both policy-makers and researchers and the health system as a whole.

## Supplementary Information

**Additional file 1: Appendix 1a.** Search strategy in PubMed. **Appendix 1b.** Search strategy in Scopus.

**Additional file 2: Appendix 2.** Interview guide.

## Data Availability

The datasets used and/or analysed during the current study are available from the corresponding author on reasonable request.

## Abbreviations

EIPM: Evidence-informed policy-making
