# Supplementary material for: Individual and institutional capacity-building for evidence-informed health policy-making in Iran: a mix of local and global evidence
Source: Health Res Policy Syst. 2022 Feb 12;20:18. doi: 10.1186/s12961-022-00816-3 (PMC8841080; doi:10.1186/s12961-022-00816-3)
Supplement: Supplementary file 1 — Additional file 1: Appendix 1a. Search strategy in PubMed. Appendix 1b. Search strategy in Scopus. [file 12961_2022_816_MOESM1_ESM.docx]

**Appendix1a: Search Strategy in PubMed**

| **Search** | **Query** | **Result** |
| --- | --- | --- |
| #1 | Decision Making, Organizational [Mesh]  The process by which decisions are made in an institution or other organization. | 10968 |
| #2 | "Policy Making"[Mesh]  The decision process by which individuals, groups or institutions establish policies pertaining to plans, programs or procedures | 23819 |
| #3 | (((((policymak*[Title/Abstract])  OR policy mak*[Title/Abstract])  OR policy-mak*[Title/Abstract])  OR decisionmak*[Title/Abstract])  OR decision mak*[Title/Abstract])  OR decision-mak*[Title/Abstract] | 153980 |
| #4 | #1 OR #2 | 34300 |
| #5 | #4 AND #3 | 5890 |
| #6 | "Evidence-Based Practice"[Mesh]  A way of providing health care that is guided by a thoughtful integration of the best available scientific knowledge with clinical expertise. This approach allows the practitioner to critically assess research data, clinical guidelines, and other information resources in order to correctly identify the clinical problem, apply the most high-quality intervention, and re-evaluate the outcome for future improvement. | 82197 |
| #7 | evidence*[Title/Abstract])  OR informe*[Title/Abstract] | 1727853 |
| #8 | #6 AND #7 | 49669 |
| #9 | #5 AND #8 | 565 |
| #10 | "evidence informed policy making"[Title/Abstract] | 51 |
| #11 | "evidence based policy making"[Title/Abstract] | 136 |
| #12 | #9 OR #10 OR #11 | 689 |
| #13 | #12 Filters: English | 673 |

**Appendix1b: Search Strategy in Scopus**

| #1 | ( TITLE-ABS-KEY ( "evidence based policy making" ) OR TITLE-ABS-KEY ( "evidence informed policy making" ) ) | 546 document results |
| --- | --- | --- |
| #2 | Limit English | 528 document results |
