# Supplementary material for: Individual and institutional capacity-building for evidence-informed health policy-making in Iran: a mix of local and global evidence
Source: Health Res Policy Syst. 2022 Feb 12;20:18. doi: 10.1186/s12961-022-00816-3 (PMC8841080; doi:10.1186/s12961-022-00816-3)
Supplement: Supplementary file 2 — Additional file 2: Appendix 2. Interview guide. [file 12961_2022_816_MOESM2_ESM.docx]

**Appendix2**

**Interview guide**

**Date and venue:**

Name and position of interviewee

1. What were Iran's interventions or empowerment programs in the past for researchers in producing and translating evidence needed for health policy?

2. What are Iran's current empowerments programs for researchers in producing and translating evidence that needed for health policy?

3. What interventions or programs need to be implemented in Iran to empower and increase the knowledge and skills of researchers in strengthening the production and translation of evidence?

3-1 What considerations should be considered when developing or implementing empowerment programs?

3-2 How we can evaluate the effectiveness of empowerment programs or interventions?

4. Which stakeholders should be considered in empowering decision makers to strengthen the use of evidence in health policy in Iran?

5. If you have other things in mind, please explain?

6. If you know another expert on the subject of research, please introduce her/his to me?

As a final question, is there any further issue you would like to add?

**Thanks for your invaluable contribution.**
